# Supplementary material for: Ctf18-RFC and DNA Pol ϵ form a stable leading strand polymerase/clamp loader complex required for normal and perturbed DNA replication
Source: Nucleic Acids Res. 2020 Jun 25;48(14):8128–45. doi: 10.1093/nar/gkaa541 (PMC7641331; doi:10.1093/nar/gkaa541)
Supplement: gkaa541_Supplemental_Files [file gkaa541_supplemental_files.zip › Macro_1.docx]

#@ File (label = "Input directory", style = "directory") srcFile

#@ String (label = "File extension", value=".dv") ext

#@ Integer (label = "cell contour channel", value=2) contour

#@ Integer (label = "GFP channel", value = 3) countchannel

#@ Integer (label = "mCherry channel", value = 4) linechannel

#@ Integer (label = "Magnification", value = 40) magnification

#@ Float (label = "Minimum distance (um)", value = 1) minimumdist

#@ Float (label = "Maximum distance (um)", value = 2.5) maximumdist

#@ Integer (label = "Minimum cell size", value = 200) min_size

#@ Integer (label = "Maximum cell size", value = 999999999) max_size

"""

count_cells_foci.py

From an open image in Fiji, generate cells ROIs from

thresholding a channel, then count the foci inside each

ROI for GPF channel and generate distances between foci in

the same ROI at mCherry channel. Finally, it saves the results

as a csv file.

"""

import os

from java.io import File

from ij import IJ, ImageStack, ImagePlus

from ij.plugin.frame import RoiManager

import math

from ij import WindowManager

from ij.measure import ResultsTable

from loci.plugins import BF

from ij.io import FileSaver

from ij.process import ImageStatistics as IS

countcells = 0

count_range = 0

cellsperfoci = {0:0, 1:0, 2:0, 3:0, "more":0}

cellsperfoci_range = {0:0, 1:0, 2:0, 3:0, "more":0}

# calculate total number of channels based on

# parameter inputs

totchannels = 2

if (linechannel > 0):

totchannels = totchannels +1

if (countchannel > 0):

totchannels = totchannels +1

# figure out which one is the relevant channel for cell shapes

# by elimination

channels = range(totchannels)

if (linechannel > 0):

channels.remove(linechannel-1)

if (countchannel > 0):

channels.remove(countchannel-1)

channels.remove(contour-1)

cellchannel=channels[0]

# camera pixel size for the DVs - relevant for pixel size

# given magnification

physicalPixel = 6.45

# returns an instance of ROI Manager (creates one if

# it doesn't exist)

def get_roi_manager(new=False):

rm = RoiManager.getInstance()

if not rm:

rm = RoiManager()

if new:

rm.runCommand("Reset")

return rm

srcDir = srcFile.getAbsolutePath()

# the the list of file names in the input directory

folders = []

filenamelist = []

for root, directories, filenames in os.walk(srcDir):

folders.append(root)

filenames.sort()

filenamelist.append(filenames)

totalcellsperfoci = []

totalcellsperfoci_range = []

totalcountcells = []

totalcount_range = []

directories = []

# skip irrelevant filenames, do stuff for relevant ones

for counter in range(len(folders)):

cellsperfoci = {0:0, 1:0, 2:0, 3:0, "more":0}

cellsperfoci_range = {0:0, 1:0, 2:0, 3:0, "more":0}

countcells = 0

count_range = 0

srcDir = folders[counter]

for filename in filenamelist[counter]:

if not filename.endswith(ext):

continue

# generate full file path for opening

print(os.path.join(srcDir, filename))

path = os.path.join(srcDir, filename)

# use the Bioformats importer to open image

IJ.run("Bio-Formats Importer", "open=" + path + " autoscale color_mode=Default view=Hyperstack stack_order=XYCZT");

image = IJ.getImage()

directory = srcDir

# calculate pixel size given camera pixel and magnification

size_x = physicalPixel/magnification

size_y = physicalPixel/magnification

# get stack from current image

stack = image.getStack()

# create empty stacks for split channels

countfoci_stack = ImageStack(image.width, image.height)

linefoci_stack = ImageStack(image.width, image.height)

# initialise variables for calculating in-focus slice

maxstddev = 0

infocus = 0

# now we go through the original image and retrieve slices to

# create substacks with split channels

for i in range(1, image.getNSlices()+1):

if (countchannel > 0):

myslice = stack.getProcessor(i*totchannels -(totchannels-countchannel))

countfoci_stack.addSlice(str(i), myslice)

if (linechannel > 0):

myslice = stack.getProcessor(i*totchannels -(totchannels-linechannel))

linefoci_stack.addSlice(str(i), myslice)

# we also calculate the standard deviation on each cell channel slice

# and update the maximum value of that

myslice = stack.getProcessor(i*totchannels -(totchannels-1 - cellchannel))

stats = IS.getStatistics(myslice)

if stats.stdDev > maxstddev:

maxstddev = stats.stdDev

infocus = i

#print(i,infocus,maxstddev)

# we set the relevant z-slice to be the maximum std dev one and get

# that "stack" (it's a single slice)

zslice = infocus

print("zslice:", zslice)

cellsproc = stack.getProcessor(zslice*totchannels -(totchannels - 1 -cellchannel))

# we create a new image from that z-slice and display it

ImagePlus("stack", cellsproc).show()

# then we close the original image

image.close()

# from now on, "image" refers to in-focus z-slice with cells

image = IJ.getImage()

# then, we create a binary image using Default thresholding

IJ.run("Make Binary", "method=Default background=Dark");

# it underestimates areas a bit, so we dilate them once and then

# separate contiguous cells using Watershed

IJ.run("Dilate");

IJ.run("Watershed");

# we establish a minimum size of 200 for something to be considered

# a cell, add cells to ROI and create an outline image

IJ.run("Analyze Particles...", "size="+str(min_size)+"-"+str(max_size)+" show=Outlines clear add");

# getting and saving outline image as a tif

image = IJ.getImage()

fs = FileSaver(image)

filepath = directory + "/" + filename + ".tif"

fs.saveAsTiff(filepath)

# now we can save the outlines and save the cell channel image.

image.changes = False

image.close()

image = IJ.getImage()

image.changes = False

image.close()

# now, we get the ROIs generated - these should be the cells

rm = get_roi_manager()

rois = rm.getRoisAsArray()

# this segment only runs if a GFP channel is present

if (countchannel > 0):

# generate image with GFP channel

ImagePlus("stack", countfoci_stack).show()

image = IJ.getImage()

# run max projection to get all foci, then close original image

IJ.run("Z Project...", "projection=[Max Intensity]");

image.close()

image = IJ.getImage()

# find maxima corresponding to foci - noise=50 has worked well

# empirically

IJ.run("Find Maxima...", "noise=750 output=List");

image.close()

# get the results table with maxima and add a "cell" column to it

rt = ResultsTable.getResultsTable()

rt.addValue("cell", 0)

countcells = countcells + len(rois)

print("countcells: "+str(countcells))

cell = 1

for roi in rois:

# this is looping over cells...

for count in range(rt.size()):

# ... and for each cell this is looping over foci

# we get XY coordinates of the foci

x = int(rt.getValue("X",count))

y = int(rt.getValue("Y", count))

# if that cell contains these coordinates, add the cell

# number as "cell" value for that foci

if roi.contains(x,y):

rt.setValue("cell", count, cell)

cell = cell + 1

# save this results table

rt.save(directory+"/"+filename+"_GFP.csv" )

print("saving at ",directory+"/"+filename+"_GFP.csv")

# create summary resulta table, with "cell" and "foci_count" columns

consol = ResultsTable()

consol.incrementCounter()

consol.addValue("cell", 0)

consol.addValue("foci_count", 0)

rowcount = 1

# loop over all cells, add cell number to the "cell" column

for count in range(cell):

consol.setValue("cell",count,count)

# loop over all foci

for count in range(rt.size()):

# get in which cell that foci is and increase the

# counter on the summary results table

currcell = int(rt.getValue("cell",count))

consol.setValue("foci_count", currcell, int(consol.getValue("foci_count", currcell))+1)

for count in range(1,cell):

foci = consol.getValue("foci_count",count)

if foci<=3:

cellsperfoci[foci] = cellsperfoci[foci] + 1

else:

cellsperfoci["more"] = cellsperfoci["more"] +1

print(cellsperfoci)

# close the results window

IJ.selectWindow("Results");

IJ.run("Close");

# this segment only runs if a mCherry channel is present

if (linechannel > 0):

# generate image with mCherry channel

ImagePlus("stack", linefoci_stack).show()

image = IJ.getImage()

# run max projection to get all foci, then close original image

IJ.run("Z Project...", "projection=[Max Intensity]");

image.close()

image = IJ.getImage()

# find maxima corresponding to foci - noise=50 has worked well

# empirically

IJ.run("Find Maxima...", "noise=750 output=List");

image.close()

# get the results table with maxima and add a "cell" column to it

rt = ResultsTable.getResultsTable()

rt.addValue("cell", 0)

cell = 1

for roi in rois:

# this is looping over cells...

for count in range(rt.size()):

# ... and for each cell this is looping over foci

# we get XY coordinates of the foci

x = int(rt.getValue("X",count))

y = int(rt.getValue("Y", count))

# if that cell contains these coordinates, add the cell

# number as "cell" value for that foci

if roi.contains(x,y):

rt.setValue("cell", count, cell)

cell = cell + 1

# add columns "dist_to" and "focus" to results table

rt.addValue("dist_to", 0)

rt.addValue("focus", 0)

# add column "is_in_range" to the summary table

consol.addValue("is_in_range", 0)

for count in range(rt.size()):

# loop over the mCherry foci

# we want to get the minimum distance between foci in the same

# cell, so we start with a very large value

mindist = 99999999

minval = -1

# cell1 is the cell where the current focus is

cell1 = rt.getValue("cell",count)

# for each focus, we loop over all foci to compare that with

for count2 in range(rt.size()):

# cell2 is the cell where the focus being compared to

# "main one" is

cell2 = rt.getValue("cell",count2)

# if we're comparing two different foci in the same cell:

if (count != count2 and cell1 == cell2):

# get their XY coordinates, calculate 2d distance

x1 = rt.getValue("X",count)*size_x

x2 = rt.getValue("X",count2)*size_x

y1 = rt.getValue("Y",count)*size_y

y2 = rt.getValue("Y",count2)*size_y

dist = math.sqrt( (x1 - x2)**2 + (y1 - y2)**2 )

# if that's the smallest distance from that "main"

# focus to another focus in the same cell, replace it

if (dist < mindist):

mindist = dist

minval = count2

# if the minimum distance from that focus to another one in

# the same cell is in the relevant range, set "is_in_range" to 1

if (mindist > minimumdist) and (mindist < maximumdist):

consol.setValue("is_in_range", int(cell1), 1)

# in the mCherry results table, set minimum distance

# and the focus to which that minimum distance is

rt.setValue("dist_to", count, mindist)

rt.setValue("focus", count, minval+1)

# save the mCherry results table

rt.save(directory+"/"+filename+"_mcherry.csv" )

# do the same foci counting procedure as for GFP

rowcount = 1

#for count in range(cell):

#consol.setValue("cell",count,count)

#consol.setValue("foci_count",count,0)

#for count in range(rt.size()):

#currcell = int(rt.getValue("cell",count))

#print(currcell, "old value", consol.getValue("foci_count", currcell), "new value", int(consol.getValue("foci_count", currcell))+1)

#consol.setValue("foci_count", currcell, int(consol.getValue("foci_count", currcell))+1)

IJ.selectWindow("Results");

IJ.run("Close");

for count in range(consol.size()):

inrange = consol.getValue("is_in_range",count)

if (inrange == 1):

count_range = count_range + 1

foci = consol.getValue("foci_count",count)

if foci<=3:

cellsperfoci_range[foci] = cellsperfoci_range[foci] + 1

else:

cellsperfoci_range["more"] = cellsperfoci_range["more"] +1

# save the summary results table

consol.save(directory+"/"+filename+"_summary.csv" )

# reset the ROI Manager, close it and go to next file (if there is one)

rm.runCommand("Reset")

rm.close()

if any(x.endswith(ext) for x in filenamelist[counter]):

fp = open(srcDir + "/total_summary.csv", "w")

fp.write("total cells, "+str(countcells)+"\n\n")

if (linechannel > 0):

fp.write("cells in range, "+str(count_range)+"\n")

fp.write("cells with foci and in range, "+str(cellsperfoci_range[1]+cellsperfoci_range[2]+cellsperfoci_range[3])+"\n\n")

for i in range(4):

fp.write("cells with "+str(i)+" foci and in range, "+str(cellsperfoci_range[i])+"\n")

fp.write("cells with more than 3 foci and in range, "+ str(cellsperfoci_range["more"])+"\n\n")

for i in range(4):

fp.write("percentage of in range cells with "+str(i)+" foci, "+str(float(cellsperfoci_range[i])/count_range)+"\n")

fp.write("percentage of in range cells with more than 3 foci, "+str(float(cellsperfoci_range["more"])/count_range)+"\n\n")

for i in range(4):

fp.write("cells with "+str(i)+" foci, "+str(cellsperfoci[i])+"\n")

fp.write("cells with more than 3 foci, "+ str(cellsperfoci["more"])+"\n\n")

for i in range(4):

fp.write("percentage of cells with "+str(i)+" foci, "+str(float(cellsperfoci[i])/countcells)+"\n")

fp.write("percentage of cells with more than 3 foci, "+str(float(cellsperfoci["more"])/countcells)+"\n\n")

fp.close()

directories.append(srcDir)

totalcountcells.append(countcells)

totalcount_range.append(count_range)

totalcellsperfoci.append(cellsperfoci)

totalcellsperfoci_range.append(cellsperfoci_range)

srcDir = srcFile.getAbsolutePath()

fp = open(srcDir + "/all_folders_summary.csv", "w")

fp.write(",")

for i in range(len(directories)):

fp.write(directories[i]+ ",")

fp.write("\n")

fp.write("total cells, ")

for i in range(len(directories)):

fp.write(str(totalcountcells[i])+ ",")

fp.write("\n\n")

if (linechannel > 0):

fp.write("cells in range, ")

for i in range(len(directories)):

fp.write(str(totalcount_range[i])+ ",")

fp.write("\n\n")

fp.write("cells with foci and in range, ")

for i in range(len(directories)):

fp.write(str(totalcellsperfoci_range[i][1]+totalcellsperfoci_range[i][2]+totalcellsperfoci_range[i][3])+ ",")

fp.write("\n\n")

for j in range(4):

fp.write("cells with "+str(j)+" foci and in range, ")

for i in range(len(directories)):

fp.write(str(totalcellsperfoci_range[i][j])+ ",")

fp.write("\n")

fp.write("cells with more than 3 foci and in range,")

for i in range(len(directories)):

fp.write(str(totalcellsperfoci_range[i]["more"])+ ",")

fp.write("\n\n")

for j in range(4):

fp.write("percentage of in range cells with "+str(j)+" foci, ")

for i in range(len(directories)):

fp.write(str(float(totalcellsperfoci_range[i][j])/totalcount_range[i])+ ",")

fp.write("\n")

fp.write("percentage of in range cells with more than 3 foci,")

for i in range(len(directories)):

fp.write(str(float(totalcellsperfoci_range[i]["more"])/totalcount_range[i])+ ",")

fp.write("\n\n")

for j in range(4):

fp.write("cells with "+str(j)+" foci, ")

for i in range(len(directories)):

fp.write(str(totalcellsperfoci[i][j])+ ",")

fp.write("\n")

fp.write("cells with more than 3 foci, ")

for i in range(len(directories)):

fp.write(str(totalcellsperfoci[i]["more"])+ ",")

fp.write("\n\n")

for j in range(4):

fp.write("percentage of cells with "+str(j)+" foci, ")

for i in range(len(directories)):

fp.write(str(float(totalcellsperfoci[i][j])/totalcountcells[i])+ ",")

fp.write("\n")

fp.write("percentage of cells with more than 3 foci,")

for i in range(len(directories)):

fp.write(str(float(totalcellsperfoci[i]["more"])/totalcountcells[i])+ ",")

fp.write("\n\n")

fp.close()
