# Supplementary material for: Ctf18-RFC and DNA Pol ϵ form a stable leading strand polymerase/clamp loader complex required for normal and perturbed DNA replication
Source: Nucleic Acids Res. 2020 Jun 25;48(14):8128–45. doi: 10.1093/nar/gkaa541 (PMC7641331; doi:10.1093/nar/gkaa541)
Supplement: gkaa541_Supplemental_Files [file gkaa541_supplemental_files.zip › Stokes et al SI Revised_no2.pdf]

Supplemental Figure Legends

Fig S1. Structural and biophysical characterization of the Ctf18-1-8/Pol2$_{CAT}$ interaction. (A) Superposition of the WH2 and WH3 domains of the C222$_1$ Ctf18-1-8/Pol2(1-528) crystal form (pdb 4oki), with Ctf8 shown in orange, Dcc1 in blue, Ctf18 in green and Pol2(1-528) in gray, and the new P3$_2$21 crystal form shown entirely in salmon. (**B**) Surface conservation calculated using the Consurf server of Pol2$_{CAT}$ and Ctf18-1-8 with the interacting surface indicated by the dashed yellow lines. Areas with least conservation are shown in cyan, and most conservation in magenta. (**C**) Comparison of subdomain movements of Pol2$_{CAT}$ (our structure and pdb 4m8o) with *E. coli* Pol II (pdb 3k5o and 3k59) (1) Structures are aligned by the NTD and EXO subdomains while subdomains indicated above are shown in red for the empty polymerase and salmon for the DNA and nucleotide-bound structure. (**D**) ITC titrations of Ctf18-1-8 in to Pol2$_{CAT}$ in the presence and absence of excess DNA substrate. (**E**) Comparison of the molecular details of the Dcc1-Pol2$_{EXO}$ interface for the Pol2$_{CAT}$/Ctf18-1-8 complex (left panel) and Pol2(1-528) complex (right panel). (**F**) Control supershift EMSA of the Ctf18-1-8 variants. 50 nM P1P2* was present in all lanes. 250 nM Ctf18-1-8 variants were loaded either with (+) or without (-) Pol2$_{CAT}$ to show that the DNA-bound bands only form when Pol2$_{CAT}$ is present and so are not due to Ctf18-1-8 binding DNA. The second lane has a high 2 μM concentration of Ctf18-1-8 to show how the Ctf18-1-8/DNA complex band would appear.

Fig S2. Cryo-EM data processing strategy.

Fig S3. Fit of cryo-EM models in maps. (**A**) Refined model and cryo-EM electron density for Class 1. (**B**) Refined model and cryo-EM electron density for Class 2. (**C**) Sidechain electron density of a well-ordered region of Class 1. (**D**) Electron density surrounding the iron-sulfur cluster of Pol2$_{CAT}$. (**E**) Rigid fit of the Pol2$_{CAT}$-nucleotide-DNA ternary complex (pdb 4m8o) into Class 1 shows that there is clearly no density for DNA or the processivity domain, which binds DNA. A reconstruction with only a spherical mask is shown to ensure that weak density is not masked out. (**F**) Comparison of Class 1 (yellow surface) with a map obtained from a sample where no DNA was present at any stage of preparation (gray mesh) showing that the structures are broadly the same.

Fig S4. Breaking the interaction between Pol2 and Ctf18-RFC. A) Analysis of the interaction between Pol ε and Ctf18-RFC during the cell cycle. The experiment was conducted as in Fig. 4A, except the cultures were not treated with formaldehyde. (**B**) Alignment of the N-terminal sequences of POL2 and orthologues in in *Schizosaccharomyces pombe*, Homo sapiens, *Mus musculus*, *Drosophila melanogaster* and *Xenopus laevis*. The sites selected for mutation and the correspondent amino acids substitution used in the screening are indicated. (**C**) Yeast-two hybrids analysis of the interaction between alleles of Pol2 (1-396) and Ctf18 (full-length). Cells were co-transformed with the plasmids as indicated. (**D**) Ctf18-RFC binding to Mcm2-7 depends on origin firing. Strains carrying auxin-inducible degrons (2) *cdc7-aid dbf4-aid* and an *MCM4-5FLAG* allele, together with a *CDC7 DBF4 Mcm4-5FLAG* and untagged control strains, were grown in YPRAF to exponential phase at 24˚C, arrested in G1, resuspended in YPGAL for 35 minutes and incubated a further hour in the presence of 0.5 mM Indole-3-acetic acid for 1 hour. Cells were then synchronously released in S phase for 30 minutes and treated with formaldehyde. Cells extracts and the proteins immunoprecipitated with anti-FLAG beads were analyzed by immunoblotting. (**E**) The binding of Ctf18-RFC at forks does not increase following fork stalling. Strains, carrying a TAP-tagged allele of MCM3 or an untagged control, were arrested in G1, and synchronously released in YPD for 30 minutes (S phase) or YPD 0.2 M HU for 90 minutes (HU). Cells were treated with formaldehyde; the cells extracts and the proteins immunoprecipitated with anti-TAP beads were analyzed by immunoblotting.

Fig S5 Analysis of the genetic interactions of the Pol2 and Ctf18-1-8 mutants. (**A**) Analysis of the meiotic progeny of the indicated heterozygotes diploids. No synthetic lethality was observed, but growth defects with *ctf18-RAA pol2-5A* were observed in combination *sgs1Δ, mre11Δ, pol32Δ and srs2Δ*. Plates were incubated at 24˚C and scanned after 3 days. (**B**) Analysis of the synthetic defects. The indicated strains were diluted 1:10 and spotted on the specified medium.

Fig S6. Checkpoint activation in response to fork stalling largely depends on the DNA damage checkpoint in mutants defective in the Pol ε/Ctf18-RFC interaction. (**A**) Analysis of checkpoint activation following replication stress. Wild type, *rad9Δ*, *rad9Δ ctf18-RAA dcc1WH3/pol2-5A* cells were arrested in G1 and synchronously released in medium containing 0.2 M HU and 0.5 mM IAA. Cells samples were collected at the indicated times and analyzed by immunoblotting for Rad53. (**B**) Deletion of *RAD9* confers synthetic sensitivity to HU in *ctf18-RAA dcc1WH3/pol2-5A* strains. The

strains were diluted 1:10 and spotted on the indicated medium. (**C**) Analysis of the dynamics of Rad53 phosphorylation in Pol2/Ctf18-RFC complex single mutants, in the presence or absence of *RAD24*. The indicated strains were treated as in panel A. (**D**) Analysis of HU sensitivity in Pol2/Ctf18-RFC complex single mutants, in the presence or absence of *RAD24*. The strains were diluted 1:10 and spotted on the indicated medium. (**E**) Analysis of double mutants for Ctf18 and Dcc1 show synthetic defects in HU sensitivity. The strains were diluted 1:10 and spotted on the indicated medium.

Table S1. Cryo-EM data collection and refinement statistics

Table S2. X-ray data collection and refinement statistics

Table S3. Yeast Strains List

Table S4. Plasmid List

Table S5. Chemicals and critical reagent list

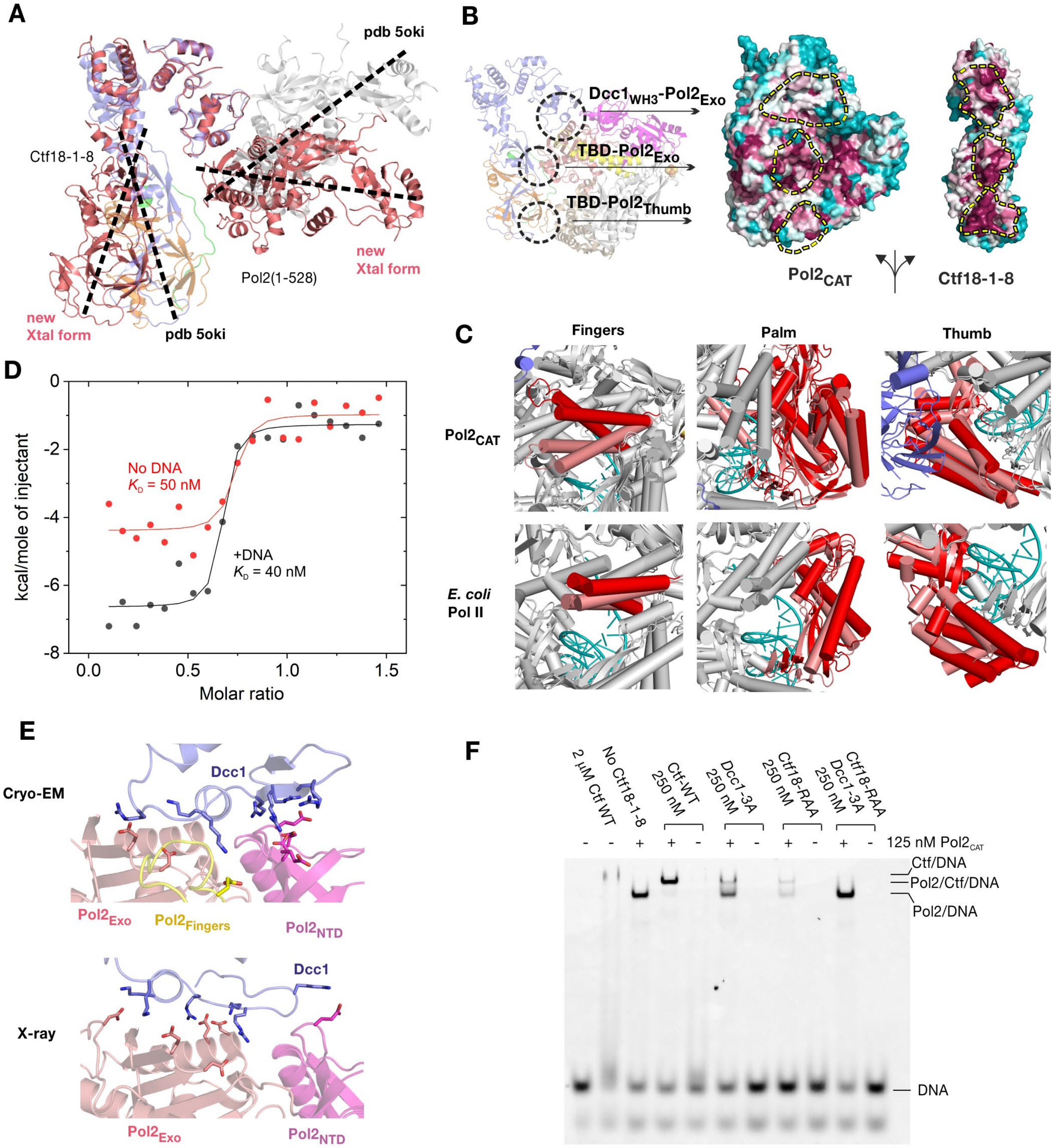
A
pdb 5oki
Ctf18-1-8
Pol2(1-528)
new Xtal form
new Xtal form
pdb 5oki
B
Dcc1WH3-Pol2Exo
TBD-Pol2Exo
TBD-Pol2Thumb
Pol2CAT
Ctf18-1-8
C
Fingers
Palm
Thumb
Pol2CAT
E. coli Pol II
D
kcal/mole of injectant
Molar ratio
No DNA
KD = 50 nM
+DNA
KD = 40 nM
E
Cryo-EM
Dcc1
Pol2Exo
Pol2Fingers
Pol2NTD
X-ray
Dcc1
Pol2Exo
Pol2NTD
F
2 µM Ctf WT
No Ctf18-1-8
Ctf-WT 250 nM
Dcc1-3A 250 nM
Ctf18-RAA 250 nM
Dcc1-3A Ctf18-RAA 250 nM
125 nM Pol2CAT
Ctf/DNA
Pol2/Ctf/DNA
Pol2/DNA
DNA

Fig. S1

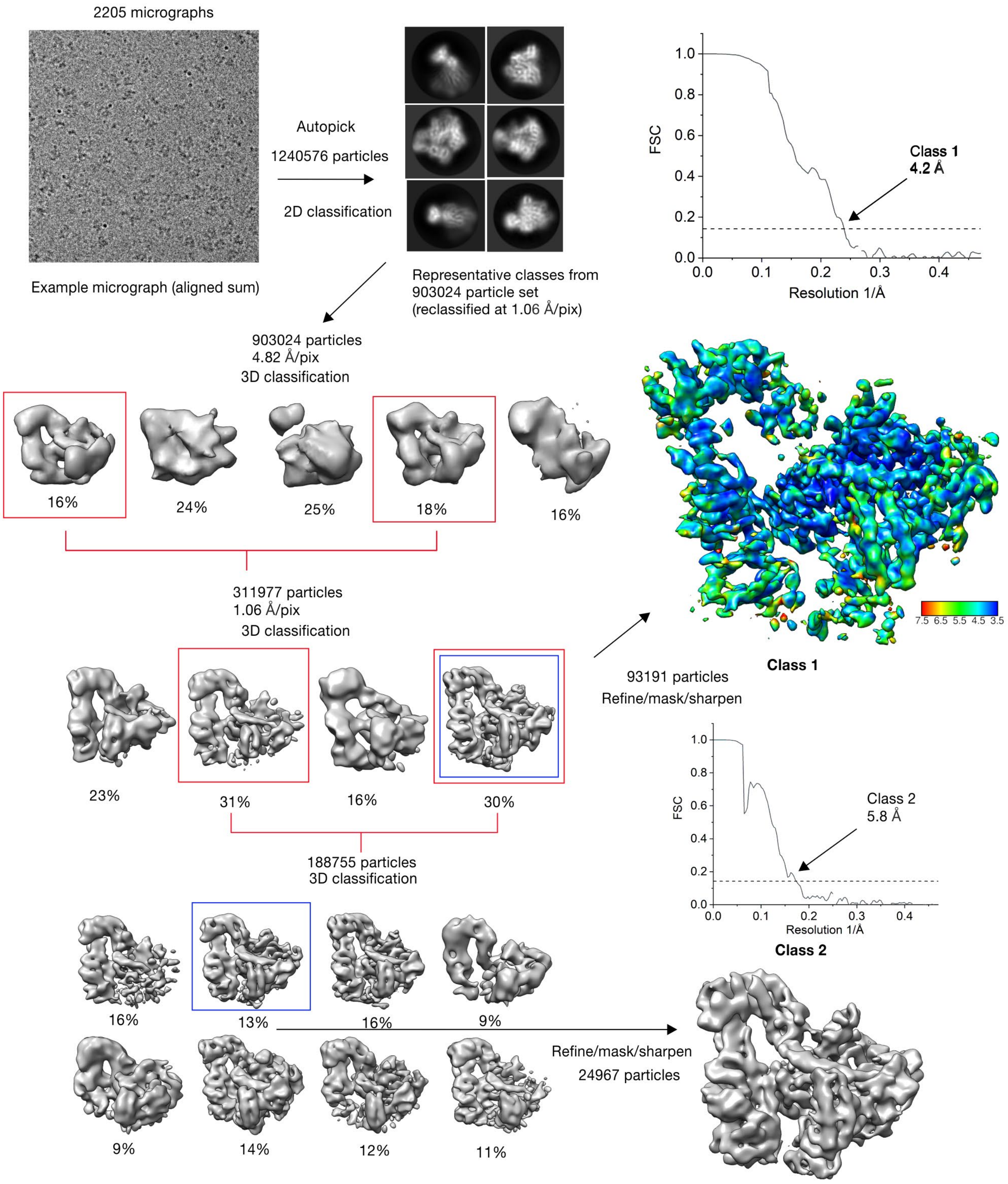
2205 micrographs
Example micrograph (aligned sum)
Autopick
1240576 particles
2D classification
Representative classes from
903024 particle set
(reclassified at 1.06 Å/pix)
FSC
Resolution 1/Å
Class 1
4.2 Å
903024 particles
4.82 Å/pix
3D classification
16%
24%
25%
18%
16%
311977 particles
1.06 Å/pix
3D classification
23%
31%
16%
30%
7.5 6.5 5.5 4.5 3.5
Class 1
93191 particles
Refine/mask/sharpen
Class 2
5.8 Å
Class 2
188755 particles
3D classification
16%
13%
16%
9%
9%
14%
12%
11%
Refine/mask/sharpen
24967 particles

Fig S2

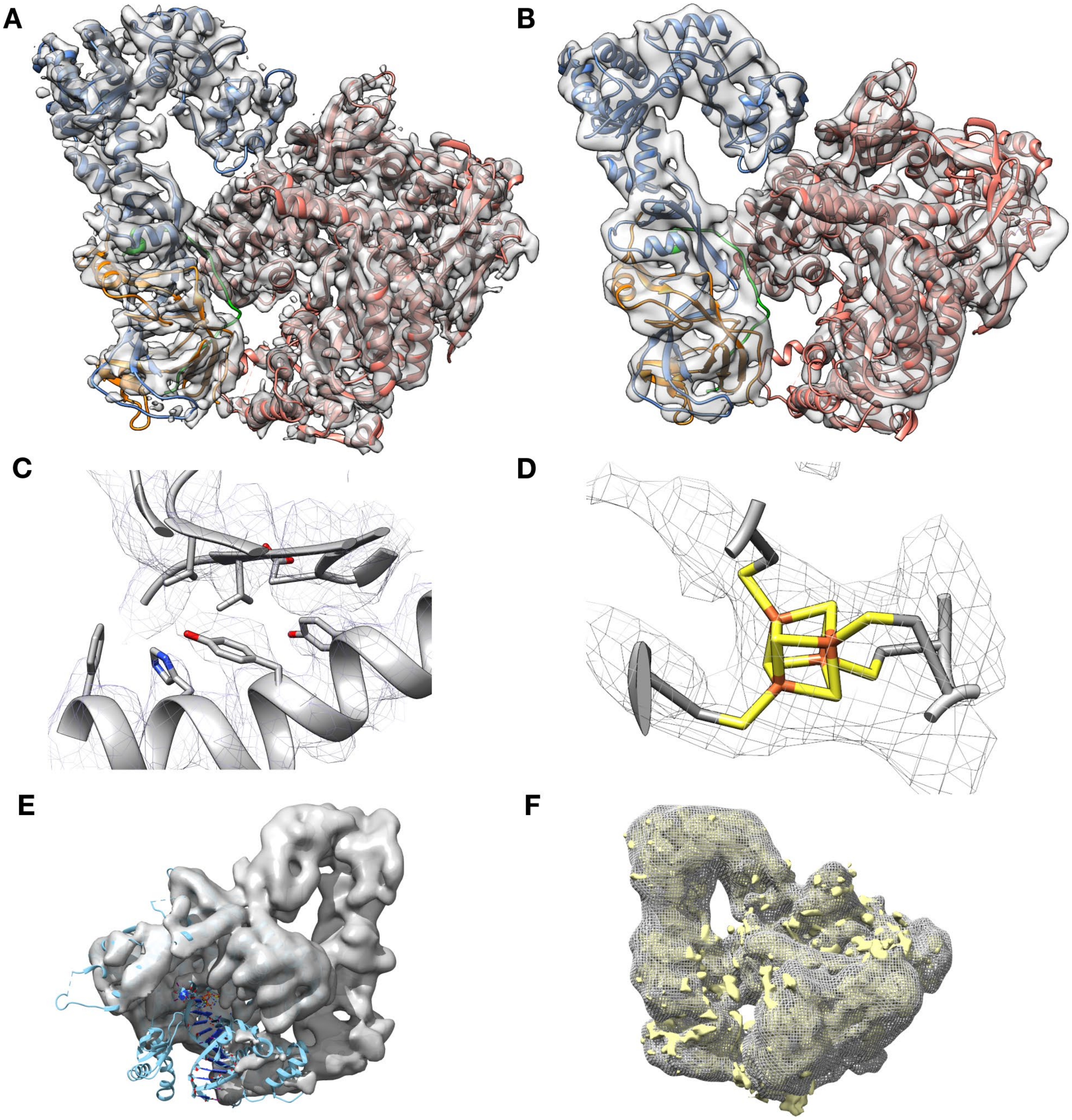

Fig S3

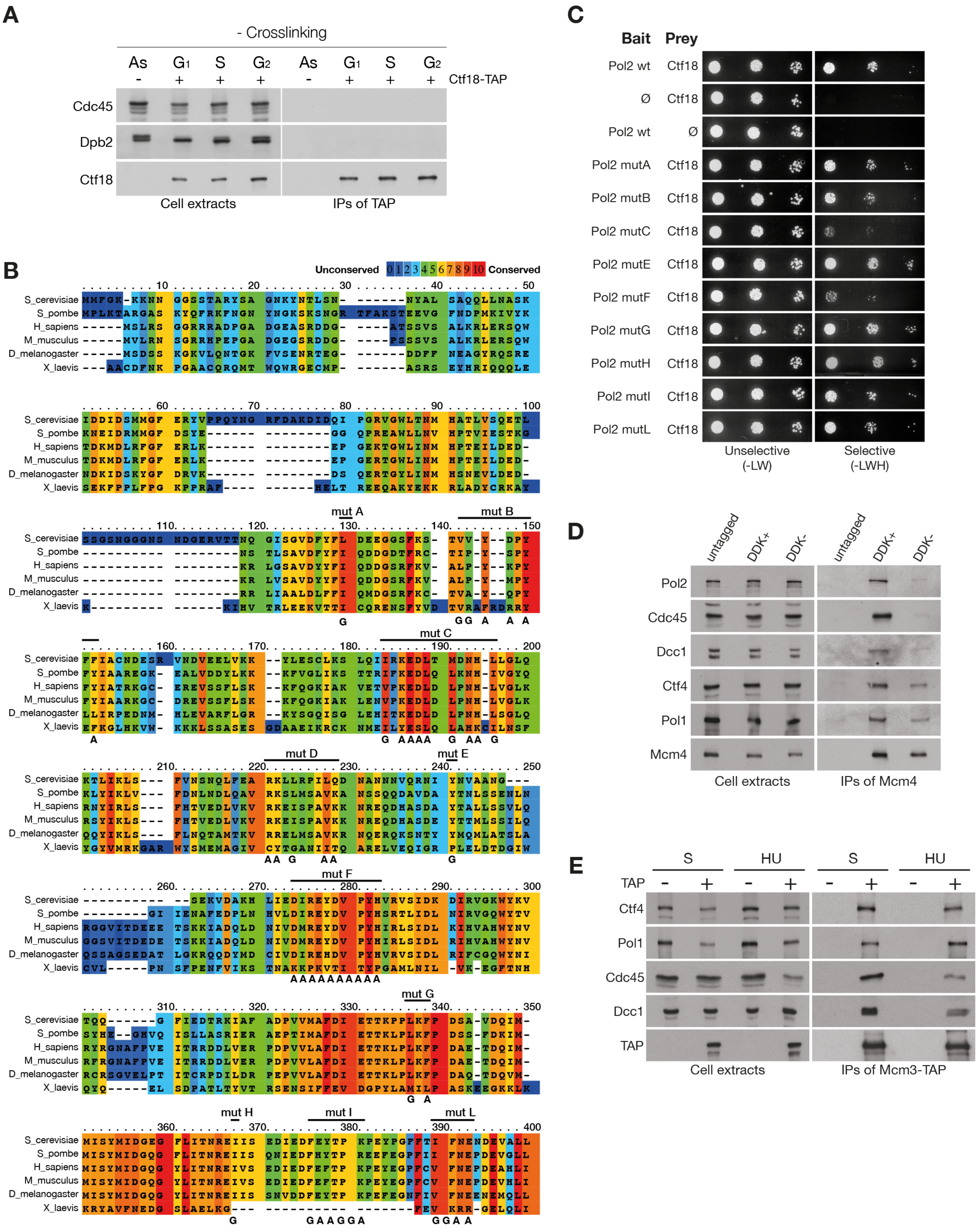
A
- Crosslinking
As
G1
S
G2
Ctf18-TAP
Cdc45
Dpb2
Ctf18
Cell extracts
IPs of TAP
B
Unconserved
Conserved
S_cerevisiae
S_pombe
H_sapiens
M_musculus
D_melanogaster
X_laevis
mut A
mut B
mut C
mut D
mut E
mut F
mut G
mut H
mut I
mut L
C
Bait
Prey
Pol2 wt
Ctf18
Ø
Pol2 mutA
Pol2 mutB
Pol2 mutC
Pol2 mutE
Pol2 mutF
Pol2 mutG
Pol2 mutH
Pol2 mutI
Pol2 mutL
Unselective
(-LW)
Selective
(-LWH)
D
untagged
DDK+
DDK-
Pol2
Cdc45
Dcc1
Ctf4
Pol1
Mcm4
Cell extracts
IPs of Mcm4
E
S
HU
TAP
Ctf4
Pol1
Cdc45
Dcc1
TAP
Cell extracts
IPs of Mcm3-TAP

Fig S4

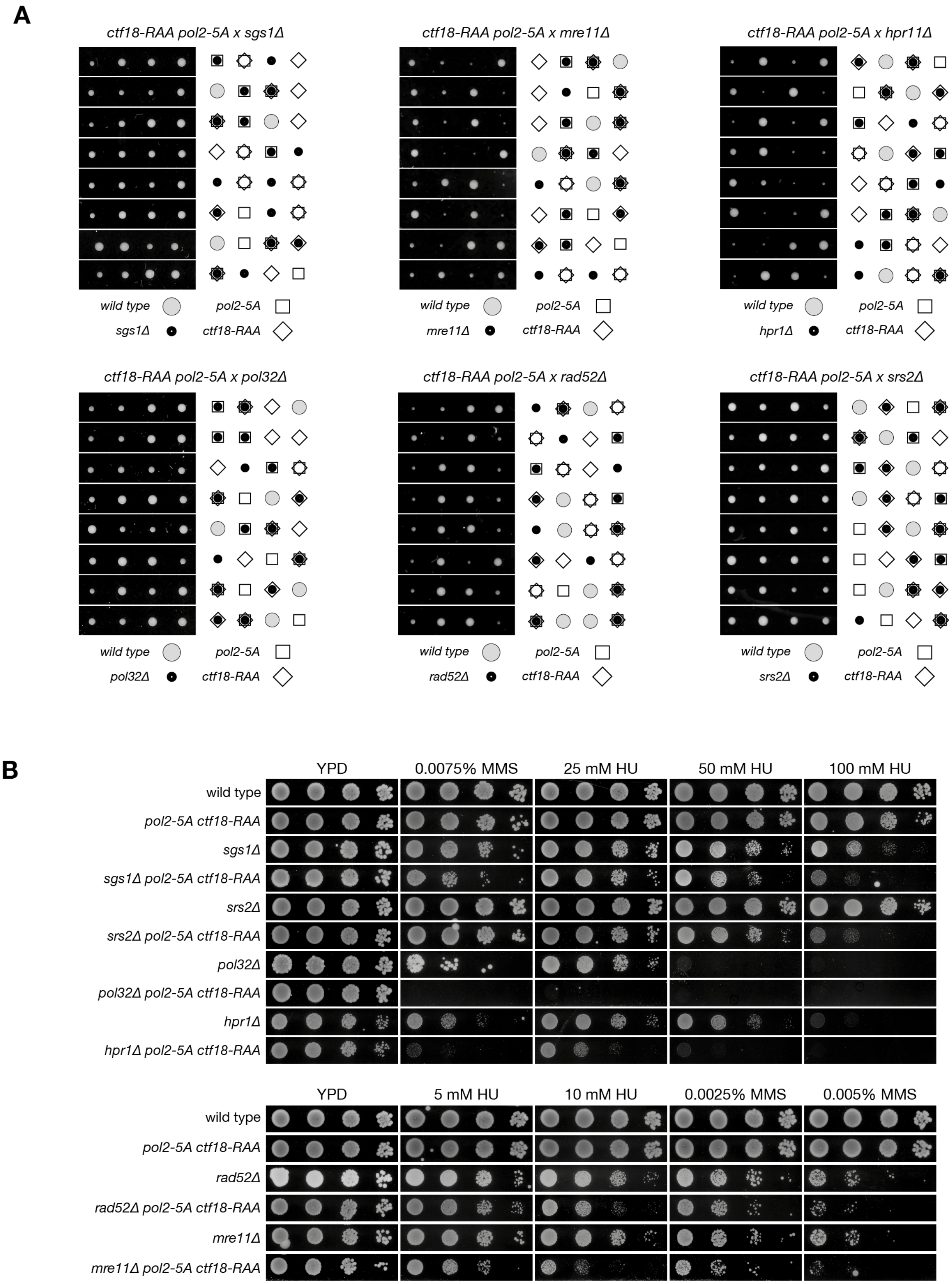
A
ctf18-RAA pol2-5A x sgs1Δ
wild type
pol2-5A
sgs1Δ
ctf18-RAA
ctf18-RAA pol2-5A x mre11Δ
wild type
pol2-5A
mre11Δ
ctf18-RAA
ctf18-RAA pol2-5A x hpr11Δ
wild type
pol2-5A
hpr1Δ
ctf18-RAA
ctf18-RAA pol2-5A x pol32Δ
wild type
pol2-5A
pol32Δ
ctf18-RAA
ctf18-RAA pol2-5A x rad52Δ
wild type
pol2-5A
rad52Δ
ctf18-RAA
ctf18-RAA pol2-5A x srs2Δ
wild type
pol2-5A
srs2Δ
ctf18-RAA
B
YPD
0.0075% MMS
25 mM HU
50 mM HU
100 mM HU
wild type
pol2-5A ctf18-RAA
sgs1Δ
sgs1Δ pol2-5A ctf18-RAA
srs2Δ
srs2Δ pol2-5A ctf18-RAA
pol32Δ
pol32Δ pol2-5A ctf18-RAA
hpr1Δ
hpr1Δ pol2-5A ctf18-RAA
YPD
5 mM HU
10 mM HU
0.0025% MMS
0.005% MMS
wild type
pol2-5A ctf18-RAA
rad52Δ
rad52Δ pol2-5A ctf18-RAA
mre11Δ
mre11Δ pol2-5A ctf18-RAA

Fig S5

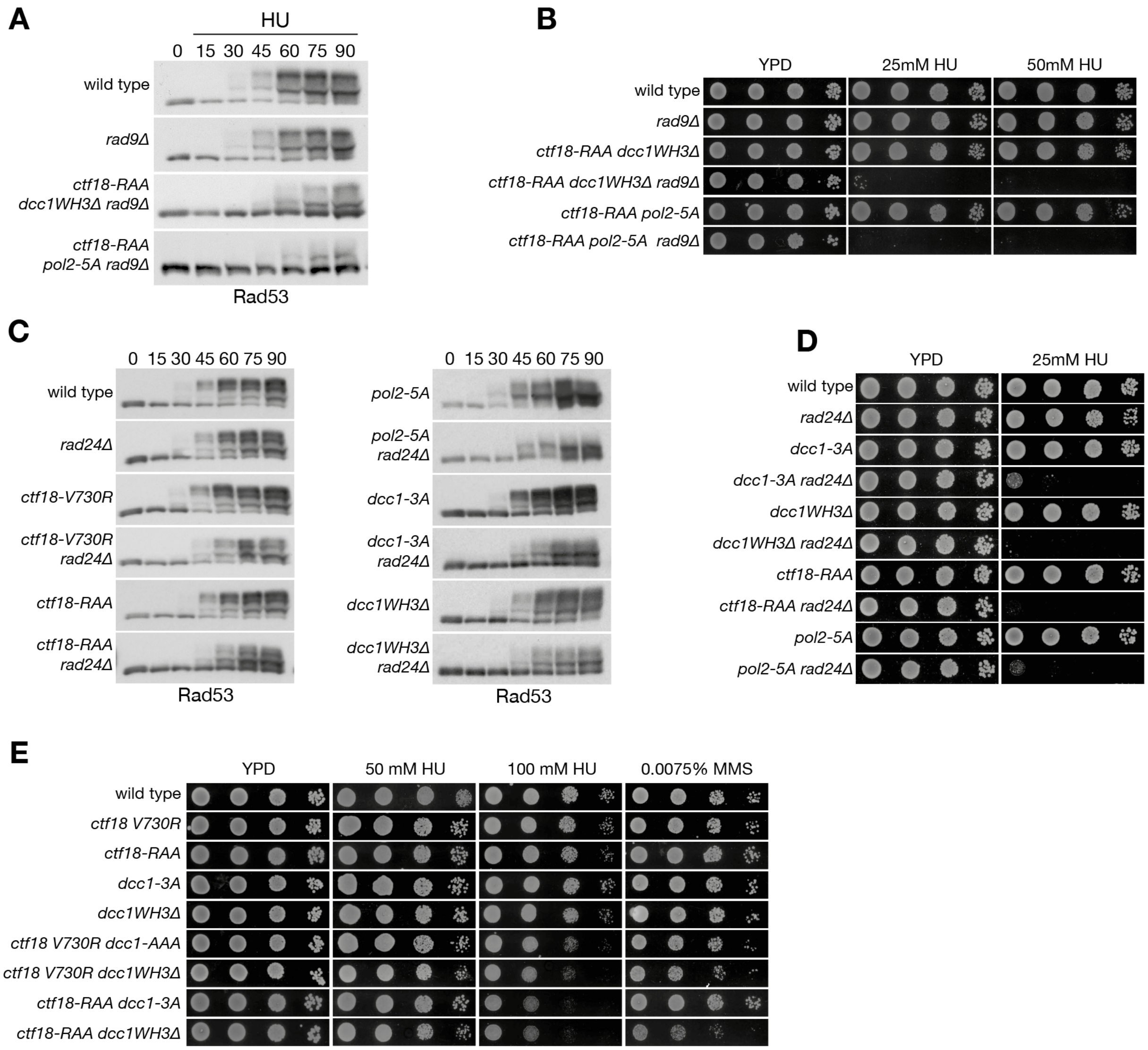
A
HU
0 15 30 45 60 75 90
wild type
rad9Δ
ctf18-RAA dcc1WH3Δ rad9Δ
ctf18-RAA pol2-5A rad9Δ
Rad53
B
YPD
25mM HU
50mM HU
wild type
rad9Δ
ctf18-RAA dcc1WH3Δ
ctf18-RAA dcc1WH3Δ rad9Δ
ctf18-RAA pol2-5A
ctf18-RAA pol2-5A rad9Δ
C
0 15 30 45 60 75 90
wild type
rad24Δ
ctf18-V730R
ctf18-V730R rad24Δ
ctf18-RAA
ctf18-RAA rad24Δ
Rad53
0 15 30 45 60 75 90
pol2-5A
pol2-5A rad24Δ
dcc1-3A
dcc1-3A rad24Δ
dcc1WH3Δ
dcc1WH3Δ rad24Δ
Rad53
D
YPD
25mM HU
wild type
rad24Δ
dcc1-3A
dcc1-3A rad24Δ
dcc1WH3Δ
dcc1WH3Δ rad24Δ
ctf18-RAA
ctf18-RAA rad24Δ
pol2-5A
pol2-5A rad24Δ
E
YPD
50 mM HU
100 mM HU
0.0075% MMS
wild type
ctf18 V730R
ctf18-RAA
dcc1-3A
dcc1WH3Δ
ctf18 V730R dcc1-AAA
ctf18 V730R dcc1WH3Δ
ctf18-RAA dcc1-3A
ctf18-RAA dcc1WH3Δ

Fig S6

Table S1. Cryo-EM data collection and refinement statistics

	$Pol2_{CAT}$-Ctf18-1-8 Class 1	$Pol2_{CAT}$-Ctf18-1-8 Class 2
**Data collection and processing**		
Magnification	75,000	75,000
Voltage (kV)	300	300
Electron exposure (e/Å^2)	60	60
Defocus range (μM)	-1.4 – -2.6	-1.4 – -2.6
Pixel size (Å)	1.06	1.06
Symmetry imposed	C1	C1
Initial particles images (no.)	1240576	1240576
Final particles images (no.)	93191	24967
Map resolution (Å)	4.2	5.8
FSC threshold	0.143	0.143
**Refinement**		
Map sharpening *B* factor (Å^2)	-143	-156
Model composition		
Non-hydrogen atoms	12459	12459
Protein residues	1539	1539
Ligands	1 (4Fe4S cluster)	1 (4Fe4S cluster)
B factors (Å^2)		
Protein	149.96	258.16
Ligand	153.81	338.56
R.m.s. deviations		
Bond lengths (Å)	0.008	0.009
Bond angles (°)	1.523	1.68
Validation		
MolProbity score	1.96	2.13
Clashscore	7.69	12.84
Poor rotamers (%)	1.88	1.88
Ramachandran plot		
Favored (%)	95.19	95.65
Allowed (%)	4.68	4.35
Disallowed (%)	0.13	0

Table S2. X-ray data collection and refinement statistics

	**Ctf18-1-8/Pol2(1-528)**
Space group	$P3_221$
**Cell dimensions**	
a, b, c (Å)	126.34, 126.34, 378.43
α, β, γ (°)	90, 90, 120
Resolution	19.97 - 6.10 (6.82 – 6.10)
R_{pim}	0.055 (1.043)
$CC_{1/2}$	0.998 (0.362)
$I/I\sigma$	9.2 (0.8)
Completeness (%)	97.2 (100)
Redundancy	9.0 (10)
**Refinement**	
Resolution	19.97 – 6.10
No. reflections (free)	8593 (840)
R_{work}/R_{free} (%)	27.5/33.3
**No. atoms**	
Protein	8613
Ligand/ion	0
Water	0
**B-factors**	
Protein	511.4
Ligand/ion	-
Water	-
**r.m.s. deviations**	
Bond lengths (Å)	0.003
Angles (°)	0.739

Table S3. Yeast Strains List

Strain name	Origin	Genotype
W303	gift from Karim Labib	*MAT**a**, ade2-1, ura3-1, his3-11,15, trp1-1, leu2-3,112, can1-100*
CS 2	gift from Karim Labib	*MAT**a**, rad9Δ (HIS3)*
CS 4	gift from Karim Labib	*MAT**a**, rad52Δ (kanMX)*
CS 11	gift from Karim Labib	*MAT**a**, sgs1Δ (kanMX)*
CS 13	gift from Karim Labib	*MAT**a**, mre11Δ (kanMX)*
CS 28	gift from Karim Labib	*MAT**a**, CTF18-TAP4 (HIS3MX), pep4Δ::URA3, ADE2*
CS 29	gift from Karim Labib	*MAT**a**, ctf18Δ (kanMX)*
CS 74	gift from Karim Labib	*MAT**a**, pep4Δ::ADE2*
CS 330	gift from Karim Labib	*MAT**a**, DPB2-TAP (kanMX), pep4Δ::ADE2*
CS 473	gift from Karim Labib	*MATα, pol32Δ (kanMX)*
CS 506	This study	*MAT**a**, rmi1Δ (HIS3MX)*
CS 795	This study	*MAT**a**, rad24Δ (hphNT)*
CS 1166	This study	*MAT**a**, pep4Δ::ADE2, MCM4-FLAG-9his (hphNT)*
CS 1167	This study	*MAT**a**, mrc1Δ (hphNT)*
CS 1203	This study	*MAT**a**, pep4Δ::ADE2, MCM3-TAP (HIS3MX)*
CS2044	This study	*MAT**a**, pol2-m170 (K.l. TRP1), CTF18-TAP4 (HIS3MX), pep4Δ::URA3, ADE2*
CS 2276	This study	*MAT**a**, hpr1Δ (kanMX)*
CS 2278	This study	*MAT**a**, mad2Δ (kanMX)*
CS 2280	This study	*MAT**a**, srs2Δ (kanMX)*
CS 2282	This study	*MAT**a**, chl1Δ (kanMX)*

CS 2312	This study	*MAT**a**, rad24Δ (hphNT), ctf18Δ (K.l.TRP1)*
CS 2365	This study	*MAT**a**, rad24Δ (hphNT), RAD52-GFP (K.l. TRP1)*
CS 2402	*(3)*	*MAT**a**, chl1-DAIA (LEU2)*
CS 2561/2	This study	*MAT**a**/α, dcc1-K364A, R367A, R380A (kanMX)*
CS 2563/4	This study	*MAT**a**/α, dcc1WH3Δ (1-318) (kanMX)*
CS 2554	gift from Karim Labib	*MAT**a**, RAD52-GFP (K.l.TRP1)*
CS 2596	This study	*MAT**a**, RAD52-GFP (K.l.TRP1), ctf18Δ (K.l.TRP1)*
CS 2783	This study	*MAT**a**, chl1-k48R (kanMX)*
CS 3267/3294	This study	*MAT**a**/α, pol2 E330A, D331A, E333A, D334A, E336A (K.l. TRP1)*
CS3272	This study	*MAT**a**, pol2 E330A, D331A, E333A, D334A, E336A (K.l. TRP1), CTF18-TAP4 (HIS3MX) pep4Δ::URA3, ADE2*
CS 3273	This study	*MAT**a**, rad24Δ (hphNT), pol2 E330A, D331A, E333A, D334A, E336A (K.l. TRP1)*
CS 3295	This study	*MAT**a**, RAD52-GFP (K.l.TRP1), pol2 E330A, D331A, E333A, D334A, E336A (K.l.TRP1)*
CS3341	This study	*MAT**a**, dcc1-K364A, R367A, R380A (kanMX), CTF18-TAP4 (HIS3MX), pep4Δ::URA3, ADE2*
CS 3343	This study	*MAT**a**, dcc1-WH3Δ (1-318) (kanMX), CTF18-TAP4 (HIS3MX), pep4Δ::URA3, ADE2*
CS 3349	This study	*MAT**a**, rad24Δ (hphNT), dcc1-K364A, R367A, R380A (kanMX)*
CS 3351	This study	*MAT**a**, rad24Δ (hphNT), dcc1WH3Δ (1-318) (kanMX)*
CS 3356/7	This study	*MAT**a**/α, ctf18-V730R, R731A, K732A (HIS3MX)*
CS3448/9	This study	*MAT**a**/α, ctf18-V730R (HIS3MX)*
CS 3479	This study	*MAT**a**, rad24Δ (hphNT), ctf18-V730R, R731A, K732A (HIS3MX)*
CS 3480/1	This study	*MAT**a**/α, dcc1-K364A, R367A, R380A (kanMX), ctf18-V730R, R731A, K732A (HIS3MX)*
CS 3482/3	This study	*MAT**a**/α, pol2 E330A, D331A, E333A, D334A, E336A (K.l. TRP1), ctf18-V730R, R731A, K732A (HIS3MX)*
CS 3502	This study	*MAT**a**, rad24Δ (hphNT), ctf18-V730R (HIS3MX)*

CS 3523	This study	*MAT**a**, ctf18-V730R, R731A, K732A (HIS3MX), DPB2-TAP (kanMX), pep4Δ::ADE2*
CS 3525	This study	*MAT**a**, ura3::3XURA3-tetO112, leu2::tetR-GFP-LEU2, SPC42-mCHERRY (kanMX)*
CS 3600	This study	*MAT**a**, ctf18-V730R (HIS3MX), DPB2-TAP (kanMX), pep4Δ::ADE2*
CS 3604	This study	*MAT**a**, ctf18-V730R, R731A, K732A (HIS3MX), MCM3-TAP (kanMX), pep4Δ::ADE2*
CS 3609	This study	*MAT**a**, ura3::3XURA3-tetO112, leu2::tetR-GFP-LEU2, SPC42-mCHERRY (kanMX), ctf18Δ (K.l.TRP1)*
CS 3759	This study	*MAT**a**, RAD52-GFP (K.l.TRP1), ctf18-V730R, R731A, K732A (HIS3MX), dcc1WH3Δ (1-318) (kanMX)*
CS 3826	This study	*MAT**a**, RAD52-GFP (K.l.TRP1), ctf18-V730R, R731A, K732A (HIS3MX), pol2 E330A, D331A, E333A, D334A, E336A (K.l.TRP1)*
CS 3827	This study	*MAT**a**, rad24Δ (hphNT), ctf18-V730R, R731A, K732A (HIS3MX), pol2-E330A, D331A, E333A, D334A, E336A (K.l. TRP1)*
CS 3829	This study	*MAT**a**, rad24Δ (hphNT), ctf18-V730R, R731A, K732A (HIS3MX), dcc1WH3Δ (1-318) (kanMX)*
CS 3834	This study	*MAT**a**, pol2-E330A, D331A, E333A, D334A, E336A (K.l. TRP1), ctf18-V730R, R731A, K732A (HIS3MX), MCM3-TAP (kanMX), pep4Δ::ADE2*
CS 3844	This study	*MAT**a**, ura3::3XURA3-tetO112, leu2::tetR-GFP-LEU2, SPC42-mCHERRY (kanMX), ctf18-V730R, R731A, K732A (HIS3MX), pol2-E330A, D331A, E333A, D334A, E336A (K.l.TRP1)*
CS 3897	This study	*MAT**a**, ura3::3XURA3-tetO112, leu2::tetR-GFP-LEU2, SPC42-mCHERRY (kanMX), ctf18-V730R, R731A, K732A (HIS3MX), dcc1WH3Δ (1-318) (kanMX)*
CS 3908	This study	*MAT**a**, dcc1WH3Δ (kanMX), ctf18-V730R, R731A, K732A (HIS3MX), MCM3-TAP (HIS3MX), pep4Δ::ADE2*
CS 3991	This study	*MAT**a**, rad9Δ (HIS3), ctf18-V730R, R731A, K732A (HIS3MX), dcc1WH3Δ (1-318) (kanMX)*
CS 3993	This study	*MAT**a**, rad9Δ (HIS3), ctf18-V730R, R731A, K732A (HIS3MX), pol2-E330A, D331A, E333A, D334A, E336A (K.l. TRP1)*
CS 4002	This study	*MAT**a**, mad2Δ (kanMX), ctf18-V730R, R731A, K732A (HIS3MX), dcc1WH3Δ (1-318) (kanMX)*
CS 4004	This study	*MAT**a**, mad2Δ (kanMX), ctf18-V730R, R731A, K732A (HIS3MX), pol2-E330A, D331A, E333A, D334A, E336A (K.l.TRP1)*
CS 4010	This study	*MAT**a, c**tf18-V730R, R731A, K732A (HIS3MX), pol2-E330A, D331A, E333A, D334A, E336A (K.l. TRP1), pol32Δ (kanMX)*
CS 4013	This study	*MAT**a, c**tf18-V730R, R731A, K732A (HIS3MX), pol2-E330A, D331A, E333A, D334A, E336A (K.l. TRP1), sgs1Δ (kanMX)*
CS 4026	This study	*MAT**a** ctf18-V730R, R731A, K732A (HIS3MX), pol2-E330A, D331A, E333A, D334A, E336A (K.l. TRP1), hpr1Δ (kanMX)*
CS 4029	This study	*Mat **a**, CTF8-TAP (HIS3MX), pep4Δ (ADE2)*
CS 4030	This study	*Mat **a**, CTF8-TAP (HIS3MX), pep4Δ (ADE2), dcc1WH3Δ (1-318) (kanMX), ctf18-V730R, R731A, K732A (HIS3MX)*

CS 4052	This study	*MAT**a**, ctf18-V730R, R731A, K732A (HIS3MX), pol2-E330A, D331A, E333A, D334A, E336A (K.l. TRP1), rad52Δ (kanMX)*
CS 4056	This study	*MAT**a**, ctf18-V730R, R731A, K732A (HIS3MX), pol2-E330A, D331A, E333A, D334A, E336A (K.l. TRP1), mre11Δ (kanMX)*
CS 4064	This study	*MAT**a**, ctf18-V730R, R731A, K732A (HIS3MX), pol2-E330A, D331A, E333A, D334A, E336A (K.l. TRP1), srs2Δ (kanMX)*
CS 4065	This study	*MAT**a**, mrc1-AID (HIS3MX), ADH1-OsTIR1 (K.L.TRP1, URA3), RAD52-GFP (K.l.TRP1)*
CS 4071	This study	*MAT**a**, MCM4-FLAG-9his (hphNT), cdc7-AID (kanMX), dbf4-AID (hphNT), GAL osTIR1-9MYC (K.l. TRP1), pep4Δ::ADE2*
CS 4098	This study	*MAT**a**, ctf18-V730R, R731A, K732A (HIS3MX), dcc1WH3Δ (kanMX), mrc1-AID (HIS3MX), ADH1-OsTIR1 (K.L.TRP1, URA3), RAD52-GFP (K.l.TRP1)*
CS 4103	This study	*MAT**a**, ctf18-V730R, R731A, K732A (HIS3MX), pol2-E330A, D331A, E333A, D334A, E336A (K.l. TRP1), mrc1-AID (HIS3MX), ADH1-OsTIR1 (K.L.TRP1, URA3), RAD52-GFP (K.l.TRP1)*
CS 4292	This study	*MAT**a**, ctf18Δ, (kanMX), mad2Δ (kanMX)*

Table S4. Plasmid List

Name	Plasmids
pCS62	pGBKT7-Pol2 NT (1-396) mutant A
pCS63	pGBKT7-Pol2 NT (1-396) mutant B
pCS64	pGBKT7-Pol2 NT (1-396) mutant C
pCS65	pGBKT7-Pol2 NT (1-396) mutant D
pCS66	pGBKT7-Pol2 NT (1-396) mutant E
pCS67	pGBKT7-Pol2 NT (1-396) mutant F
pCS68	pGBKT7-Pol2 NT (1-396) mutant G
pCS69	pGBKT7-Pol2 NT (1-396) mutant H
pCS70	pGBKT7-Pol2 NT (1-396) mutant I
pCS71	pGBKT7-Pol2 NT (1-396) mutant L
pCS85	pEGFP-C1 POLE1 m134
pCS86	pEGFP-C1 POLE1 wt
pCS88	pEGFP-N1 POLE1 m134
pCS91	EGFP-N1 POLE1 wt
pCS160	pEGFP-C1 acidic
pCS162	pEGFP-N1 acidic
pCS94	pGADT7-CHTF18 wild type
pCS97	pGBKT7-POLE1 1-450
pGDP33	pGBKT7-POLE1 1-450 m134
pKL559	pGADT7

pKL560	pGBKT7
pLG1	pGADT7-CTF18 wild type (4)
pLG55	pGBKT7-Pol2 NT (1-396) wild type (4)

Table S5. Chemicals and critical reagent list

Chemical and critical reagents	Manufacturer
HU	Molekula LTD (10872383 | 127-07-1)
MMS	Sigma-Aldrich (129925)
Bleomycin	VWR (203401)
Benomyl (PESTANAL (R) , analytical standard)	Sigma-Aldrich (45339)
Yeast extract (Bacto™)	Appleton Woods
Peptone (Oxoid)	Fisher Scientific (10404013)
Yeast nitrogen base (Difco™)	Appleton Woods
Alpha factor	Pepceuticals
Sigma protease inhibitor cocktail	Sigma-Aldrich (P8215)
EDTA-free Complete Protease Inhibitor Cocktail	Roche (11873580001)
Glycerophosphate	ALFA (AESAR) (L03425.22)
Dynabeads (M-270 Epoxy)	Invitrogen
PierceTM Universal Nuclease for Cell Lysis	ThermoFisher (88700)
Amersham ECL Western blotting reagents	GE Healthcare (RPN 2209)
Anti-mouse-HRP	New England Biolabs (UK) LTD (7076S)
Anti-sheep-HRP	Sigma (S1265)
Anti-rabbit-HRP	Cell Signaling (7074S)
Anti-FLAG M2	Sigma (F1804)

Anti-Dcc1	gift from Karim Labib
Anti-Pol2	gift from Karim Labib
Anti-Pol1	gift from Karim Labib
Anti-Cdc45	gift from Karim Labib
Anti-Ctf4	gift from Karim Labib
Anti-Psf1	gift from Karim Labib
Anti-Rad53	Abcam PLC [EL7.E1] (ab166859)
Anti-POLE1	Abcam PLC (ab226848)
Anti-CHTF18	Abcam PLC (ab220288)

Supplemental References

1. Wang, F. and Yang, W. (2009) Structural insight into translesion synthesis by DNA Pol II. *Cell*, **139**, 1279-1289.
2. Nishimura, K., Fukagawa, T., Takisawa, H., Kakimoto, T. and Kanemaki, M. (2009) An auxin-based degron system for the rapid depletion of proteins in nonplant cells. *Nat Methods*, **6**, 917-922.
3. Samora, C., P., Saksouk, J., Goswami , P., Wade, B., O., Singleton, M., R., Bates, P., A., Lengronne, A., Costa, A. and Uhlmann, F. (2016) Ctf4 links DNA replication with sister chromatid cohesion establishment by recruiting the Chl1 helicase to the replisome. *Molecular Cell*, **63**, 371–384.
4. García-Rodríguez, L., J., De Piccoli, G., Marchesi, V., Jones, R., C ., Edmondson, R., D . and Labib, K. (2015) A conserved Polϵ binding module in Ctf18-RFC is required for S-phase checkpoint activation downstream of Mec1. *Nucleic Acids Research*, **14**, 8830-8838.
